# Convergence of Light and ABA Signaling on the *ABI5* Promoter

**DOI:** 10.1371/journal.pgen.1004197

**Published:** 2014-02-27

**Authors:** Dongqing Xu, Jigang Li, Sreeramaiah N. Gangappa, Chamari Hettiarachchi, Fang Lin, Mats X. Andersson, Yan Jiang, Xing Wang Deng, Magnus Holm

**Affiliations:** 1 Department of Biological and Environmental Sciences, Gothenburg University, Gothenburg, Sweden; 2 Peking-Yale Joint Center for Plant Molecular Genetics and Agro-Biotechnology, State Key Laboratory of Protein and Plant Gene Research, School of Life Sciences, Peking University, Beijing, China; 3 State Key Laboratory of Plant Physiology and Biochemistry, College of Biological Sciences, China Agricultural University, Beijing, China; 4 Department of Molecular, Cellular, and Developmental Biology, Yale University, New Haven, Connecticut, United States of America; Peking University, China

## Abstract

Light is one of the most important environmental cues regulating multiple aspects of plant growth and development, and abscisic acid (ABA) is a plant hormone that plays important roles during many phases of the plant life cycle and in plants' responses to various environmental stresses. How plants integrate the external light signal with endogenous ABA pathway for better adaptation and survival remains poorly understood. Here, we show that BBX21 (also known as SALT TOLERANCE HOMOLOG 2), a B-box (BBX) protein previously shown to positively regulate seedling photomorphogenesis, is also involved in ABA signaling. Our genetic data show that BBX21 may act upstream of several *ABA INSENSITIVE (ABI)* genes and ELONGATED HYPOCOTYL 5 (HY5) in ABA control of seed germination. Previous studies showed that HY5 acts as a direct activator of *ABI5* expression, and that BBX21 interacts with HY5. We further demonstrate that BBX21 negatively regulates *ABI5* expression by interfering with HY5 binding to the *ABI5* promoter. In addition, ABI5 was shown to directly activate its own expression, whereas BBX21 negatively regulates this activity by directly interacting with ABI5. Together, our study indicates that BBX21 coordinates with HY5 and ABI5 on the *ABI5* promoter and that these transcriptional regulators work in concert to integrate light and ABA signaling in *Arabidopsis thaliana*.

## Author Summary

Many factors such as light, phytohormone abscisic acid (ABA), etc., regulate multiple developmental processes throughout the plants' life cycle. Light promotes seed germination and ABA maintains seed dormancy. However, little is known about how light and ABA signaling pathways interact with each other. It was previously reported that *Arabidopsis* HY5, a well-known bZIP transcription factor involved in promoting seedling photomorphogenesis, is involved in ABA signaling by directly activating *ABI5* expression. Here, we report that the B-box protein BBX21 negatively regulates *ABI5* expression by interfering with HY5 binding to the *ABI5* promoter. Interestingly, *ABI5* was shown to directly bind to its own promoter and activate its expression, whereas BBX21 also negatively regulates this activity by interacting with ABI5. Together, our study shows that light and ABA signaling pathways converge on the *ABI5* promoter, on which BBX21 acts as a negative regulator of *ABI5* expression.

## Introduction

Light is of utmost importance to sessile plants, not only as the primary energy source for photosynthesis, but also as an environmental signal regulating their growth and development throughout their life cycle. Light signals are perceived by several families of photoreceptors in plants, including phytochromes [primarily absorb red (R) and far-red (FR)], cryptochromes and phototropins [absorb blue (B) and ultraviolet-A (UV-A)], and a recently characterized ultraviolet-B (UV-B) photoreceptor UV RESISTANCE LOCUS 8 (UVR8) [1]. Upon light irradiation, multiple photo-activated photoreceptors (except for UVR8) repress the activity of CONSTITUTIVELY PHOTOMORPHOGENIC 1 (COP1), a central repressor of photomorphogenesis induced by visible-light [2]. COP1 is a conserved RING finger E3 ubiquitin ligase targeting several photomorphogenesis-promoting proteins for degradation, including ELONGATED HYPOCOTYL 5 (HY5) [3] and HY5 HOMOLOG (HYH) [4]. HY5, a constitutively-nuclear bZIP protein, is the first known and most extensively studied transcription factor involved in promoting photomorphogenesis under a wide spectrum of wavelengths, including FR, R, B, and UV-B [3], [5], [6], [7]. It was shown that the abundance of HY5 protein is directly correlated with the extent of photomorphogenic development [3]. Recent chromatin immunoprecipitation (ChIP)-chip studies revealed that HY5 binds directly to a large number of genomic sites, mainly at the promoter regions of annotated genes [8]–[9].

Several recent studies demonstrated that B-box (BBX) family proteins act as important transcriptional regulators in response to light and circadian cues. The BBX family represents a subgroup of zinc finger proteins that contain one or more N-terminal zinc binding BBX domains [10]. In *Arabidopsis thaliana*, the BBX family consists of 32 proteins, and can be divided into five subfamilies according to its protein sequence [10]. CONSTANS (CO), a key player in the regulation of flowering by photoperiod, was the first identified member of BBX family in *Arabidopsis* [10]–[11]. CO and its five other similar proteins belong to Subfamily I, whose members all contain two BBX domains in the N terminus and a CCT domain in the C terminus [10]. However, for the eight members in Subfamily IV (BBX18 to BBX25), each BBX protein contains two BBX domains in the N terminus but lack the CCT domain [10], [12]. Interestingly, in this subfamily, BBX21/SALT TOLERANCE HOMOLOG 2 (STH2) and BBX22/STH3/LIGHT-REGULATED ZINC FINGER PROTEIN 1 (LZF1) function as positive regulators of photomorphogenesis, whereas BBX18/DBB1a, BBX19/DBB1b, BBX24/SALT TOLERANCE (STO) and BBX25/STH1 act as negative regulators of photomorphogenesis [13]–[22]. Notably, BBX21, BBX22, BBX24 and BBX25 were all shown to physically interact with HY5, and the B-box domains of these BBX proteins and the bZIP domain of HY5 mediate their interactions [13]–[17], [20]. In addition, it was shown that HY5 promotes the expression of *BBX22* by directly binding to its promoter [13], whereas BBX24 and BBX25 repress *BBX22* expression by interfering with HY5 transcriptional activity [17].

The plant hormone abscisic acid (ABA) regulates several aspects of plant growth and development, plays an essential role in plants' adaptive responses to environmental stress, and is a key regulator of stomatal aperture [23]–[24]. Genetic studies in *Arabidopsis* have identified several factors that participate in ABA signal transduction, including ABA INSENSITIVE 1 (ABI1) to ABI5. ABI1 and ABI2, group A type protein phosphatases 2C (PP2Cs), negatively regulate SNF1-related protein kinase 2 (SnRK2) proteins which phosphorylate downstream targets such as various AREB/ABFs [25]–[26]. ABI3, ABI4, and ABI5 belong to three distinct classes of transcription factors, i.e. the basic B3, AP2/ERF, and the basic leucine zipper (bZIP) families, respectively, and share overlapping functions in ABA signaling during seed germination and early seedling development [27]–[29]. It was reported that ABI5 acts downstream of ABI3 and is involved in postgerminational developmental arrest and repression of germination [30]–[32]. Interestingly, a recent study showed that HY5 directly binds to the promoter of *ABI5* and is required for the expression of *ABI5* and ABI5-targeted genes [33]. In addition, HY5 binding to the *ABI5* promoter is enhanced by ABA treatment, and overexpression of ABI5 restores ABA sensitivity in *hy5* mutants and leads to enhanced light responses in the wild type plants [33]. Thus, HY5 regulation of *ABI5* expression represents an integration of light and ABA signaling during seed germination and early seedling development.

Accumulating evidence indicates that BBX proteins work in concert with HY5 in regulating photomorphogenesis [14]–[17], [20]. Since HY5 directly activates *ABI5* expression, it is interesting to investigate whether BBX proteins are also involved in ABA signaling. In this paper, we report that *bbx21* mutants showed hypersensitive phenotypes to ABA and NaCl treatments, and our genetic data suggest that BBX21 may act upstream of several *ABI* genes and HY5. Furthermore, we show that BBX21 negatively regulates *ABI5* expression by interfering with HY5 binding to the *ABI5* promoter. In addition, ABI5 binds to its own promoter and activate its expression, whereas BBX21 negatively regulates this activity. Taken together, our data reveal a complicated but delicate control of crosstalk between light and ABA signaling in *Arabidopsis*.

## Results

### BBX21 is involved in ABA signaling

**Figure 1:**
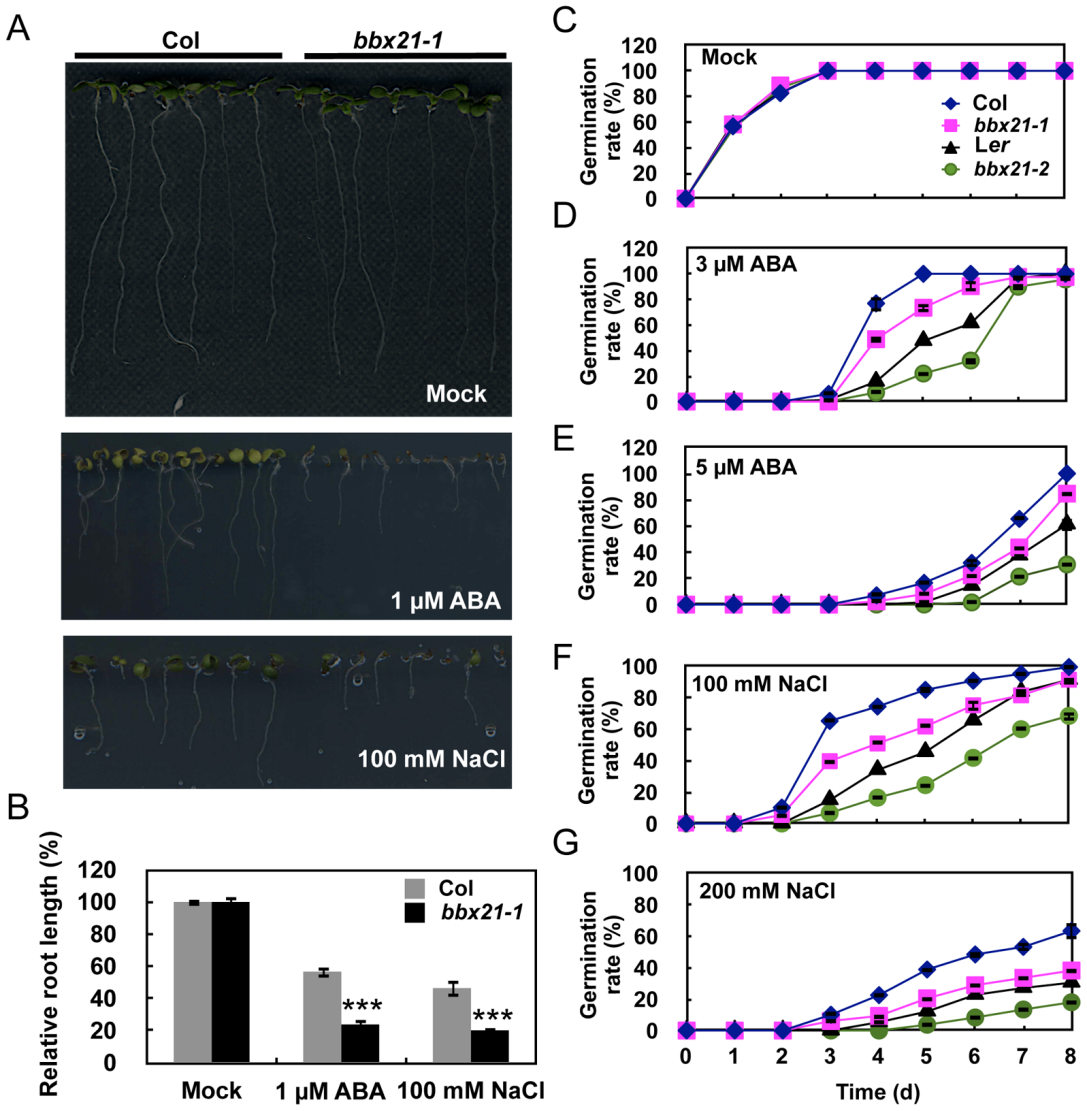
The *bbx21* mutants are hypersensitive to ABA and NaCl during germination. (A) ABA and NaCl hypersensitivity phenotypes of *bbx21-1* mutants. All seedlings were grown vertically on germination medium (GM) plates with or without 1 µM ABA or 100 mM NaCl for 7 d after stratification. (B) Root lengths of the wild type (Col) and *bbx21-1* grown on GM with or without 1 µM ABA or 100 mM NaCl. Relative root lengths compared with those of Col grown on GM plates are indicated. Values are means ± SD (n = 20). ***P<0.001 (Student's *t* test) for the differences between *bbx21-1* and the wild type. (C–G) Germination rates of the *bbx21-1* (Col) and *bbx21-2* (L*er*) mutants and their corresponding wild type controls under mock (C) and various concentrations of ABA (D and E) or NaCl (F and G) treatments. Germination rate was determined from three replicates (>150 seeds from each genotype), and error bars represent SD.

To investigate whether BBX21 is involved in ABA signaling, we examined the responses of *bbx21-1* mutants (Col background) [15] to ABA treatment. We observed that *bbx21-1* mutants were significantly more sensitive to 1 µM ABA treatment than was the wild type (Figure 1A, 1B). In addition, *bbx21-1* mutants showed more sensitivity to 100 mM NaCl than did the wild type (Figure 1A, 1B). These observations suggest that BBX21 may be involved in ABA responses.

To examine whether the shorter roots observed in *bbx21* mutants resulted from delayed germination or the inhibition of primary root growth, we compared the germination rate of wild type and *bbx21* mutant seeds grown under various ABA concentrations. As shown in Figure 1C–1E, the germination rate of both *bbx21-1* and *bbx21-2* (L*er* background) [15] mutants was lower than that of the wild type at all tested ABA concentrations (i.e. 1, 3 and 5 µM ABA). Consistently, the germination rate of both *bbx21* mutants was also lower than that of the wild type plants under treatments of 100 and 200 mM NaCl (Figure 1F, 1G). However, no difference in root length was observed between the wild type and *bbx21* mutants after they were transferred to media containing ABA or NaCl (data not shown), indicating that the short root length of *bbx21* mutants shown in Figure 1A and 1B appeared to be predominantly due to delayed germination rather than inhibition of root growth.

**Figure 2:**
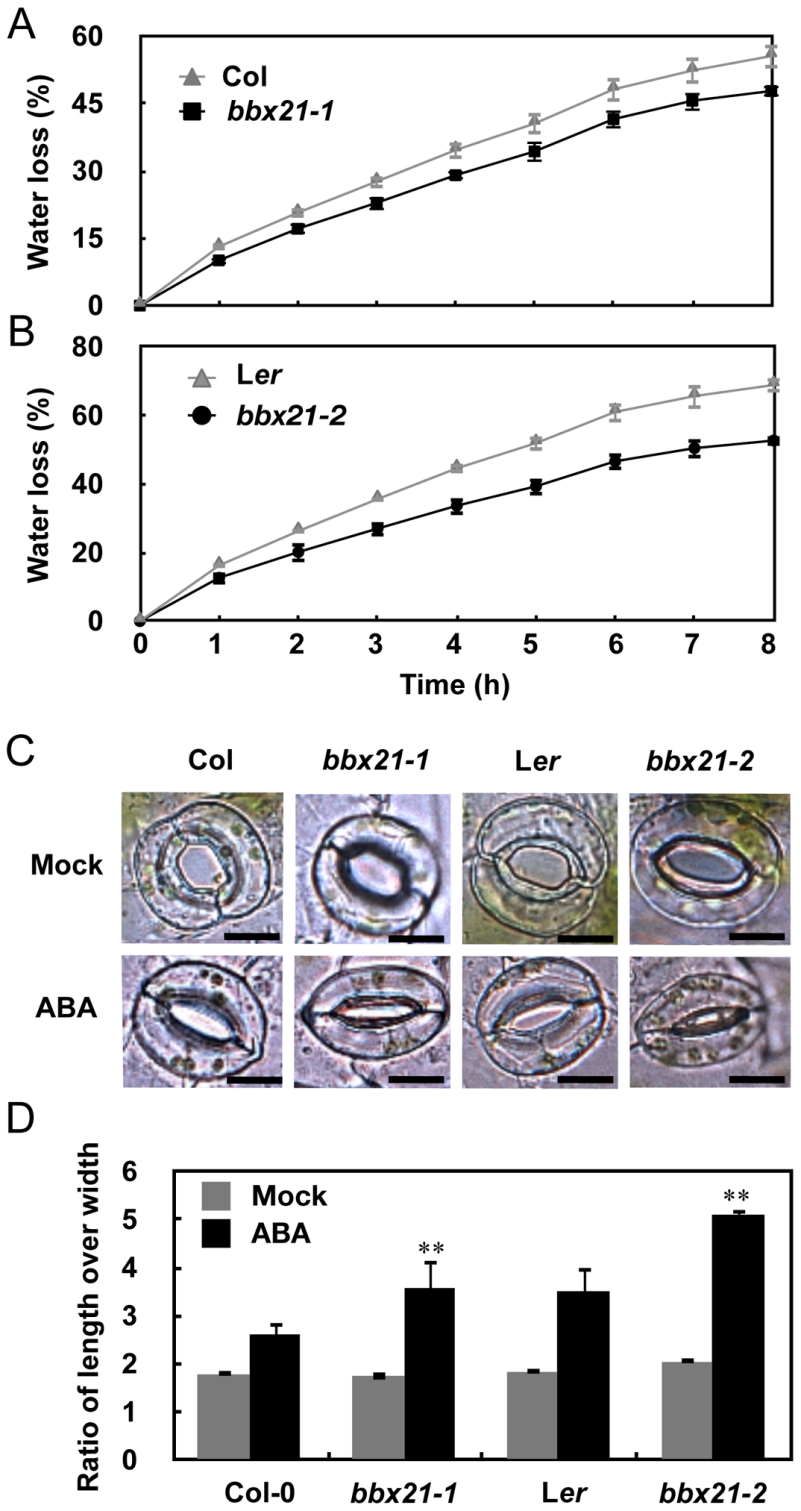
The *bbx21* mutants lost water more slowly than did the wild type plants. (A–B) Water loss from the detached leaves of the wild type, *bbx21-1*, and *bbx21-2*. Results are means of three replicates, and error bars represent SD. (C) Representative images of stomata of wild type and *bbx21* mutant plants treated with mock or 0.5 µM ABA. Bar = 10 µm. (D) Ratios of stomatal aperture length to width. Three independent experiments were performed with similar results. Data were from one experiment with 30 stomata cells from leaves of three different plants with triple replicates. Data are means ±SEs. **P<0.01 (Student's *t* test) for the differences between *bbx21* and the wild type.

Since ABA plays an important role in dehydration tolerance through stomatal closure [23]–[24], we also examined whether BBX21 is involved in ABA-mediated dehydration tolerance. As shown in Figure 2A and 2B, water loss assays showed that the leaves of both *bbx21* mutants lost water more slowly than did the wild type plants after detachment, indicating that BBX21 is involved in dehydration tolerance in adult plants. Because water loss primarily depends on stomatal regulation, we then compared the stomatal apertures of 3-w-old plants of *bbx21* mutants and their respective wild type. The epidermal peels from rosette leaves at the same developmental stage were observed with a microscope. As shown in Figure 2C, whereas no differences were observed in stomatal apertures between wild type and *bbx21* mutant plants under mock treatment, wild type plants displayed wider stomatal apertures than did the *bbx21* mutants under the treatment of 0.5 µM ABA. These observations were consistent with the statistical analysis shown in Figure 2D, in which the ratio of stomatal length to width, indicating the degree of stomatal closure, was calculated for each plant. Taken together, our data showed that mutation in *BBX21* leads to increased sensitivity to ABA.

**Figure 3:**
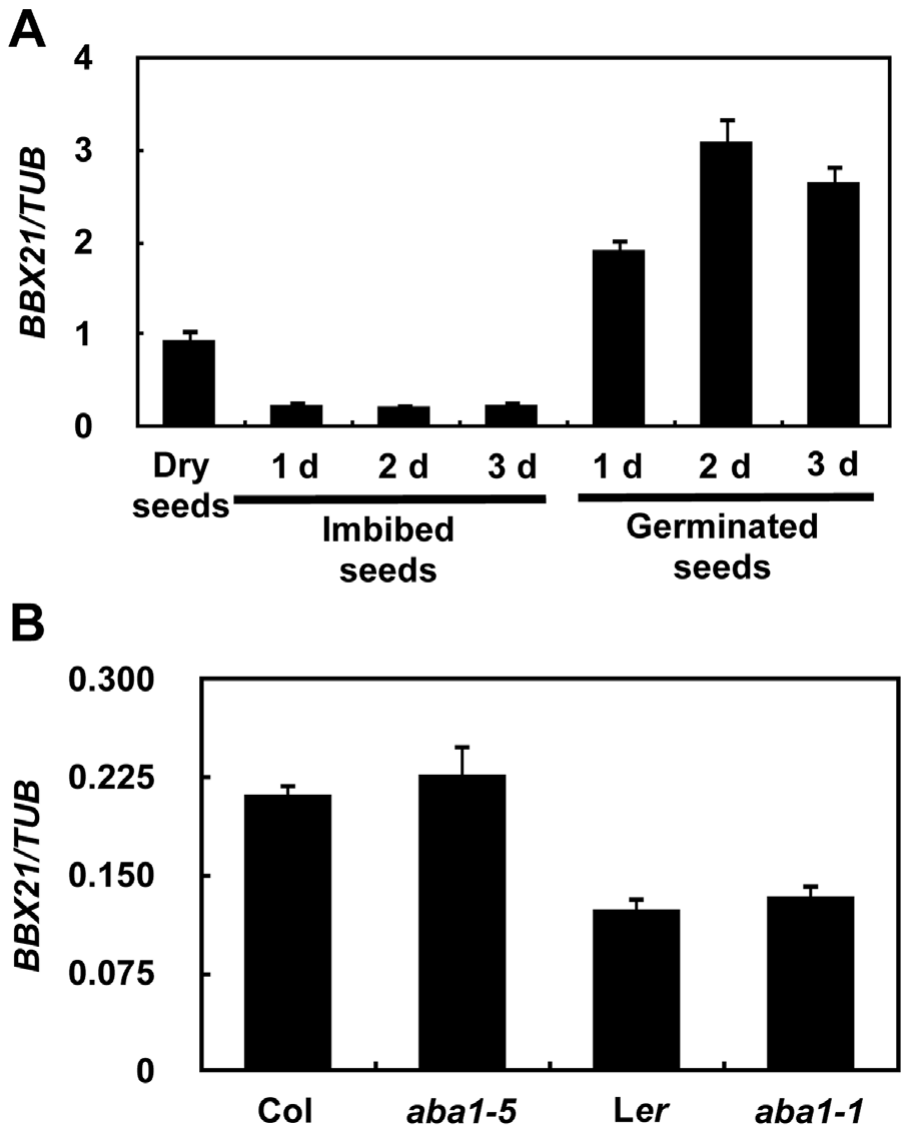
Expression pattern of *BBX21*. (**A**) **Expression pattern of BBX21 during germination.** (B) The expression levels of *BBX21* in the wild type and *aba1* mutants. Data are means of three independent experiments, and error bars represent SD.

To rule out the possibility that BBX21 may be involved in ABA biosynthesis instead of ABA signaling, we measured the ABA levels in dry seeds and 2-d-old seedlings of wild type and *bbx21* mutants using liquid chromatography–mass spectrometry (LC-MS). Our data show that there is no significant difference in ABA levels between wild type and the *bbx21* mutants in these two developmental stages, and as expected, ABA levels were dramatically reduced in 2-d-old seedlings relative to dry seeds in both wild type and the *bbx21* mutants (Figure S1). We further examined the expression pattern of *BBX21* during seed germination. *BBX21* is expressed in dry seeds of the wild type, but its expression is repressed in imbibed seeds (Figure 3A). Interestingly, after the seeds were germinated, *BBX21* expression was again induced, and reached its peak 2 days after germination (Figure 3A). In addition, *BBX21* expression was not regulated by endogenous ABA levels, because in two *aba1* alleles, which are impaired in ABA biosynthesis, the expression level of *BBX21* was indistinguishable from those of the wild type (Figure 3B). Taken together, our data indicate that BBX21 is involved in ABA signaling in *Arabidopsis*.

### BBX21 may act upstream of ABI2, ABI3, ABI4, ABI5 and HY5

**Figure 4:**
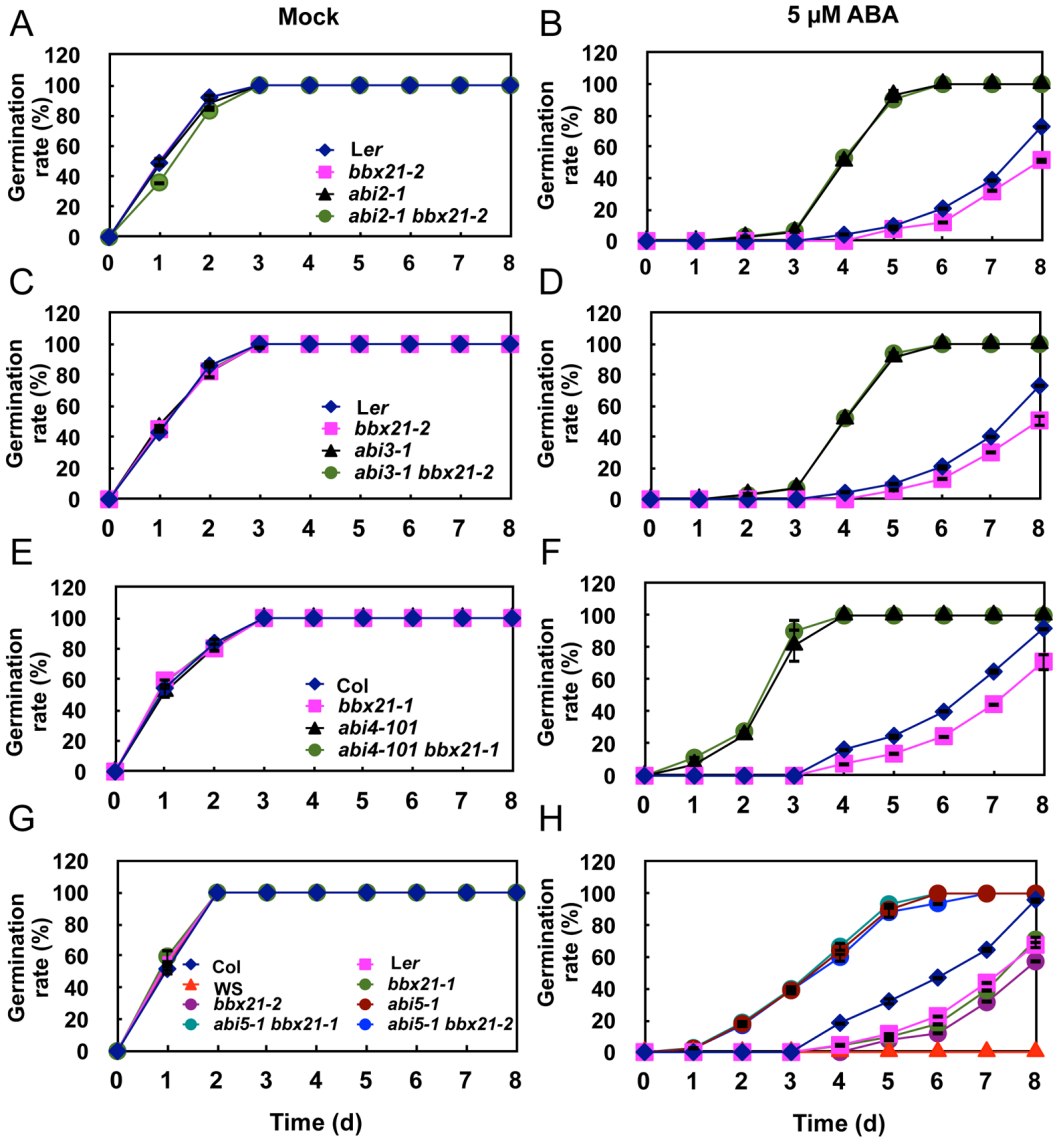
Germination rates of *bbx21, ab*i and *abi bbx21* mutants. Germination rates of the *bbx21, abi2, abi3, abi4, abi5* and *abi bbx21* mutants under mock (A, C, E and G) and 5 µM of ABA (B, D, F and H) treatments. Germination rate was determined from three replicates (>150 seeds from each genotype), and error bars represent SD.

To determine the possible roles of BBX21 in ABA signal transduction, we generated double mutants of *bbx21* with several ABA insensitive mutants, including *abi2-1* [34], *abi3-1* [35], *abi4-101* [36] and *abi5-1* [27]. The *abi3-1*, *abi4-101* and *abi5-1* mutants are null alleles of ABI3, ABI4 and ABI5, respectively, whereas *abi2-1* contains a dominant mutation (G168D) in the PP2C domain of ABI2. We analyzed the germination rates of the respective homozygous double mutants treated with 5 µM ABA. Surprisingly, we observed that all of the double mutants displayed ABA insensitivity similar as that observed in the respective *abi* single mutants (Figure 4). These observations suggest that BBX21 may act upstream of ABI2, ABI3, ABI4 and ABI5 in the ABA signaling pathway.

**Figure 5:**
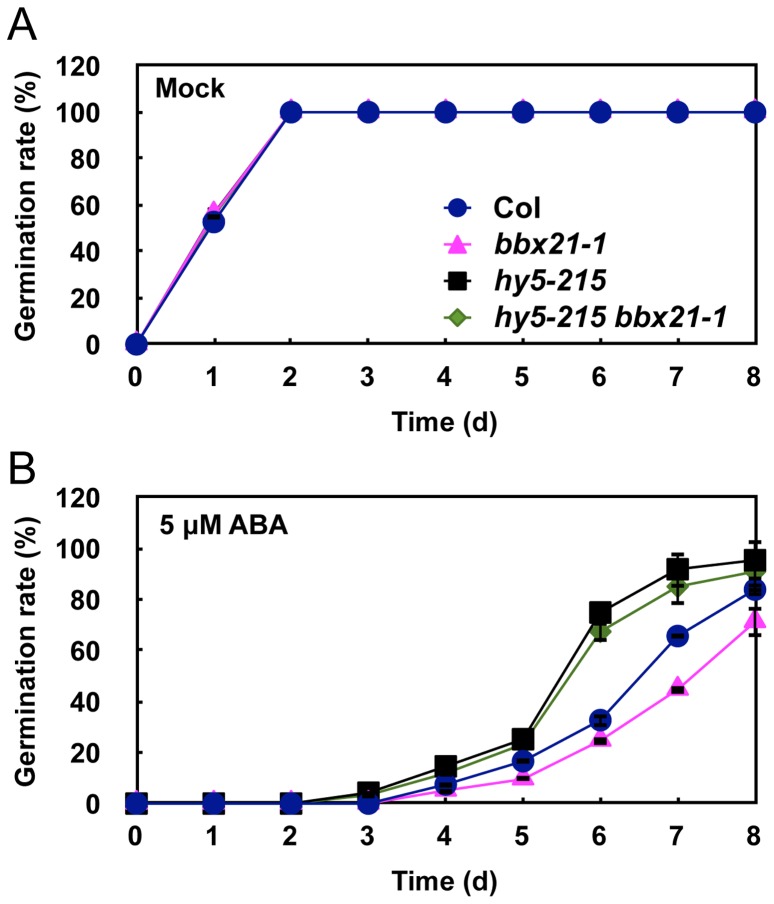
The *hy5 bbx21* mutants are insensitive to ABA during germination. Germination rates of Col, *bbx21-1, hy5-215* and *hy5-215 bbx21-1* mutants under mock (A) and 5 µM ABA (B) treatments. Error bars represent SD.

It was reported that HY5 also mediates ABA responses in seed germination [33]. Therefore, we sought to investigate the genetic relationship between HY5 and BBX21 in ABA control of seed germination. To this end, we first confirmed that all three *hy5* alleles, *hy5-215* (Col-0), *hy5-ks50* (WS) and *hy5-1* (L*er*), displayed reduced ABA sensitivity in seed germination (Figure S2). Then, we examined the germination rates of *hy5-215 bbx21-1* double mutants treated with 5 µM ABA. Our data showed that *hy5-215 bbx21-1* exhibited ABA insensitivity during germination, almost indistinguishable from that of *hy5-215* (Figure 5). These data indicate that *hy5* is epistatic to *bbx21*, and that the ABA insensitivity of *bbx21* during seed germination is dependent on a functional HY5 protein.

### BBX21 regulates the expression of *ABI3* and *ABI5*

Since BBX21 acts as a transcriptional regulator, we were interested to investigate whether BBX21 could be involved in regulating the expression of ABA signaling intermediates, such as several *ABI* genes. Therefore, we examined the expression of *ABI1* to *ABI5* in 2-d-old wild type and *bbx21* mutant plants treated with mock or 0.5 µM ABA. Our real time quantitative RT-PCR (qRT-PCR) assays showed that BBX21 negatively regulates the expression of *ABI3* and *ABI5* under both mock and ABA treatments, but is not involved in regulation of other *ABI* genes (Figure S3). Interestingly, this regulation mainly occurred in Col background, whereas in L*er* background, it was less significant or even absent (Figure S3).

**Figure 6:**
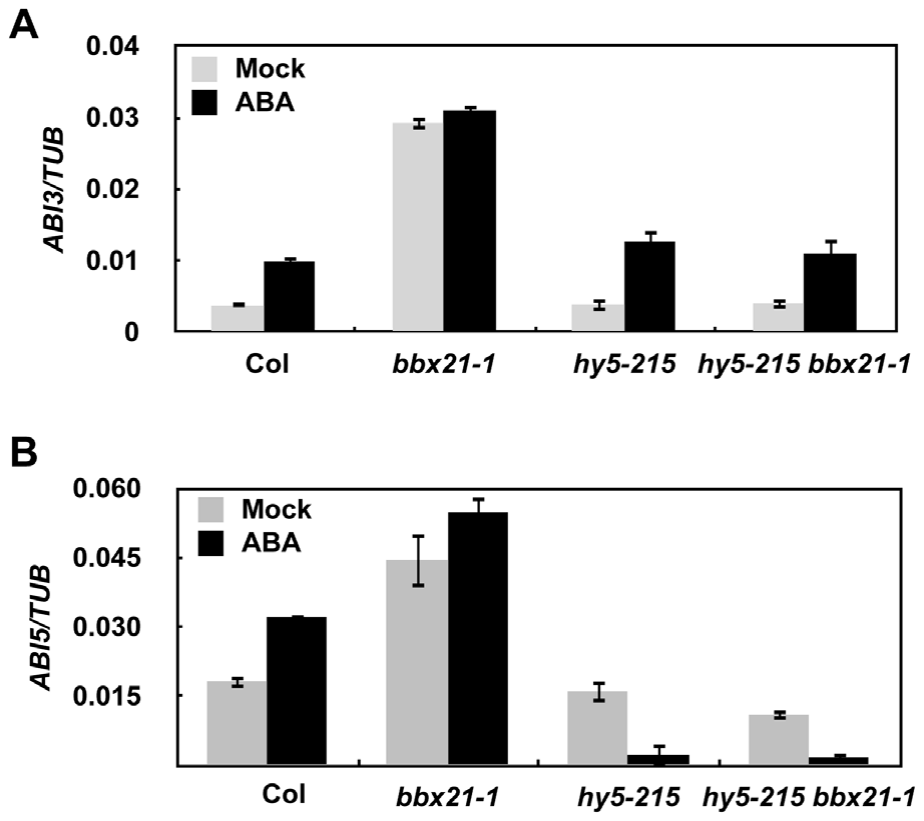
BBX21 regulates the expression of *ABI3* and *ABI5*. Expression levels of *ABI3* (A) and *ABI5* (B) in 2-d-old germinating seeds of Col, *hy5-215, bbx21-1*, and *hy5-215 bbx21-1* mutant treated with mock or 0.5 µM ABA.

It was previously shown that HY5 directly binds to the promoter of *ABI5* and activate its gene expression [33]. To investigate whether HY5 is also involved in regulating *ABI3* expression, and to better understand how BBX21 works in concert with HY5 in regulating *ABI5*, we examined the expression of *ABI3* and *ABI5* in *hy5, bbx21*, and *hy5/bbx21* mutants treated with mock or ABA. Our qRT-PCR results show that HY5 may not be involved in regulating *ABI3* expression (Figure 6A). However, BBX21 is dependent on functional HY5 to negatively regulate the expression of *ABI3* and *ABI5* (Figure 6), consistent with our genetic evidence that *hy5* is epistatic to *bbx21* (Figure 5).

### BBX21 negatively regulates *ABI5* expression by interfering with HY5 binding to the *ABI5* promoter

**Figure 7:**
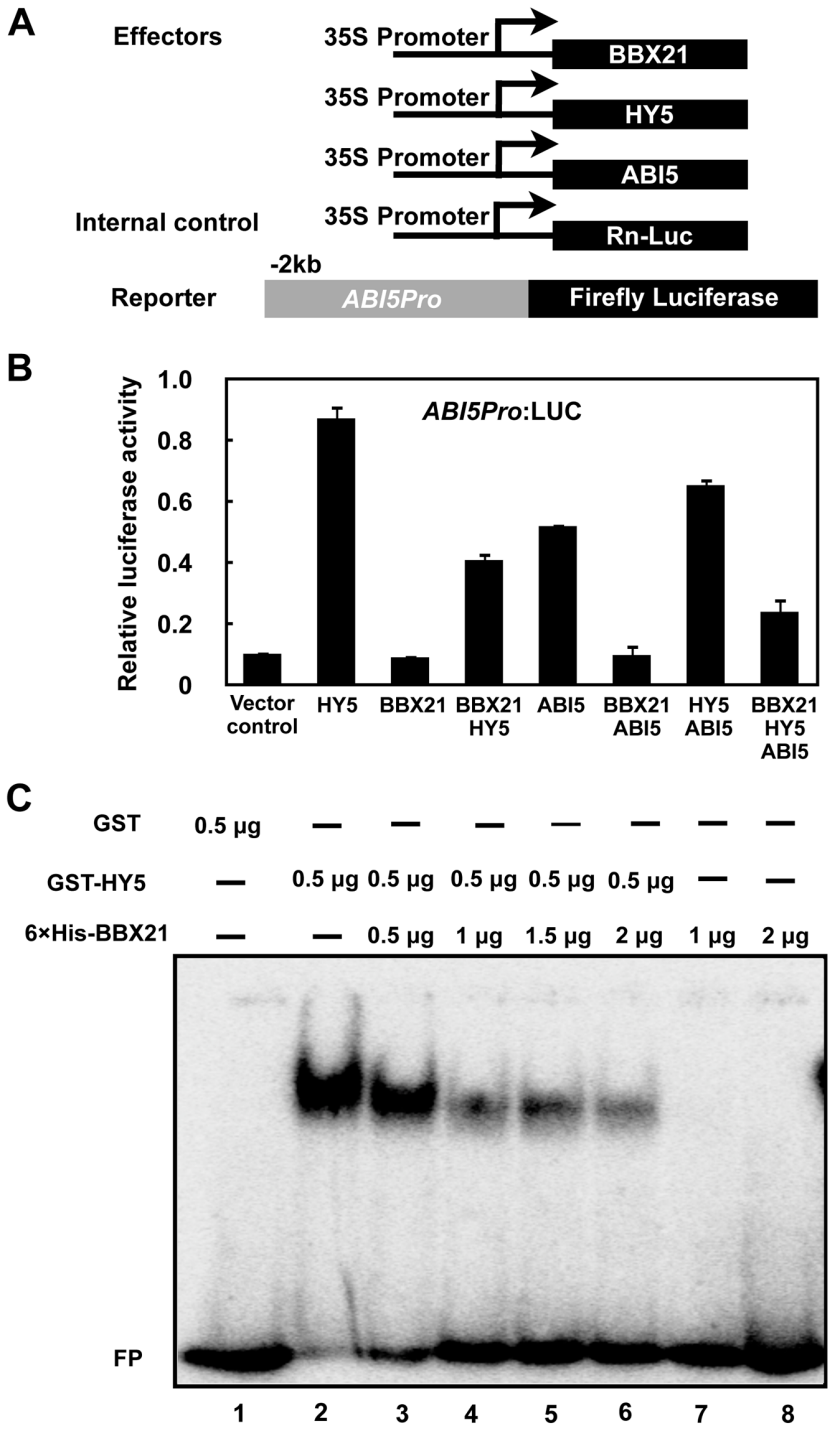
BBX21 negatively regulates *ABI5* expression by interfering with HY5 binding to the *ABI5* promoter. (A) Schematic representation of constructs used in the transient transfection assays in *Arabidopsis* protoplasts. Arrows after the 35S promoter indicate the transcription start site, and −2 kb indicate the length of the *ABI5* promoter that was fused to the firefly luciferase to create the reporter construct. (B) Activation of *ABI5p:LUC* by indicated combinations of proteins. Error bars represent SE (*n* = 3). (C) EMSAs showing that increasing amounts of His-BBX21 protein (lanes 2 to 6) decreased the binding of GST-HY5 to the *ABI*5 promoter.

To unravel the molecular mechanisms by which BBX21 negatively regulates *ABI5* expression, we performed transient transfection assays in *Arabidopsis* protoplasts using a reporter in which the luciferase reporter gene (*LUC*) was under the control of a 2-kb promoter fragment of *ABI*5. The *ABI5p:LUC* reporter was transfected into *Arabidopsis* mesophyll protoplasts along with *35S:RnLUC* (an internal control of transformation efficiency) and the effectors (*35S:BBX21, 35S:HY5*, or both) (Figure 7A). As shown in Figure 7B, HY5 alone robustly activate the *ABI5* promoter, consistent with a previous report that HY5 is a direct activator of *ABI5* [33]. BBX21 alone was unable to either activate or repress the *ABI5* promoter; however, co-expression of BBX21 with HY5 obviously decreased the *ABI5* promoter activity (Figure 7B), implying that BBX21 represses the activation of the *ABI5* promoter by interfering with HY5 action.

To further investigate how BBX21 negatively regulates *ABI5* expression, we expressed GST (glutathione *S*-transferase), GST-HY5, and 6×His-tagged BBX21 proteins in *Escherichia coli*, and performed electrophoretic mobility shift assays (EMSAs) to test whether BBX21 could affect HY5 binding to the *ABI5* promoter. As shown in Figure 7C, GST-HY5, but not GST alone, bound to the promoter fragment of *ABI5*. The 6×His-BBX21 protein was unable to bind to the *ABI5* promoter. However, increasing amounts of 6×His-BBX21 obviously decreased the GST-HY5 binding to the ABI5 promoter (Figure 7C), indicating that the physical interaction between BBX21 and HY5 prevents HY5 from binding to the ABI5 promoter. Taken together, our data demonstrated that BBX21 negatively regulates ABI5 expression by interfering with HY5 binding to the ABI5 promoter.

### ABI5 directly activates its own expression, whereas BBX21 negatively regulates this activity by directly interacting with ABI5

**Figure 8:**
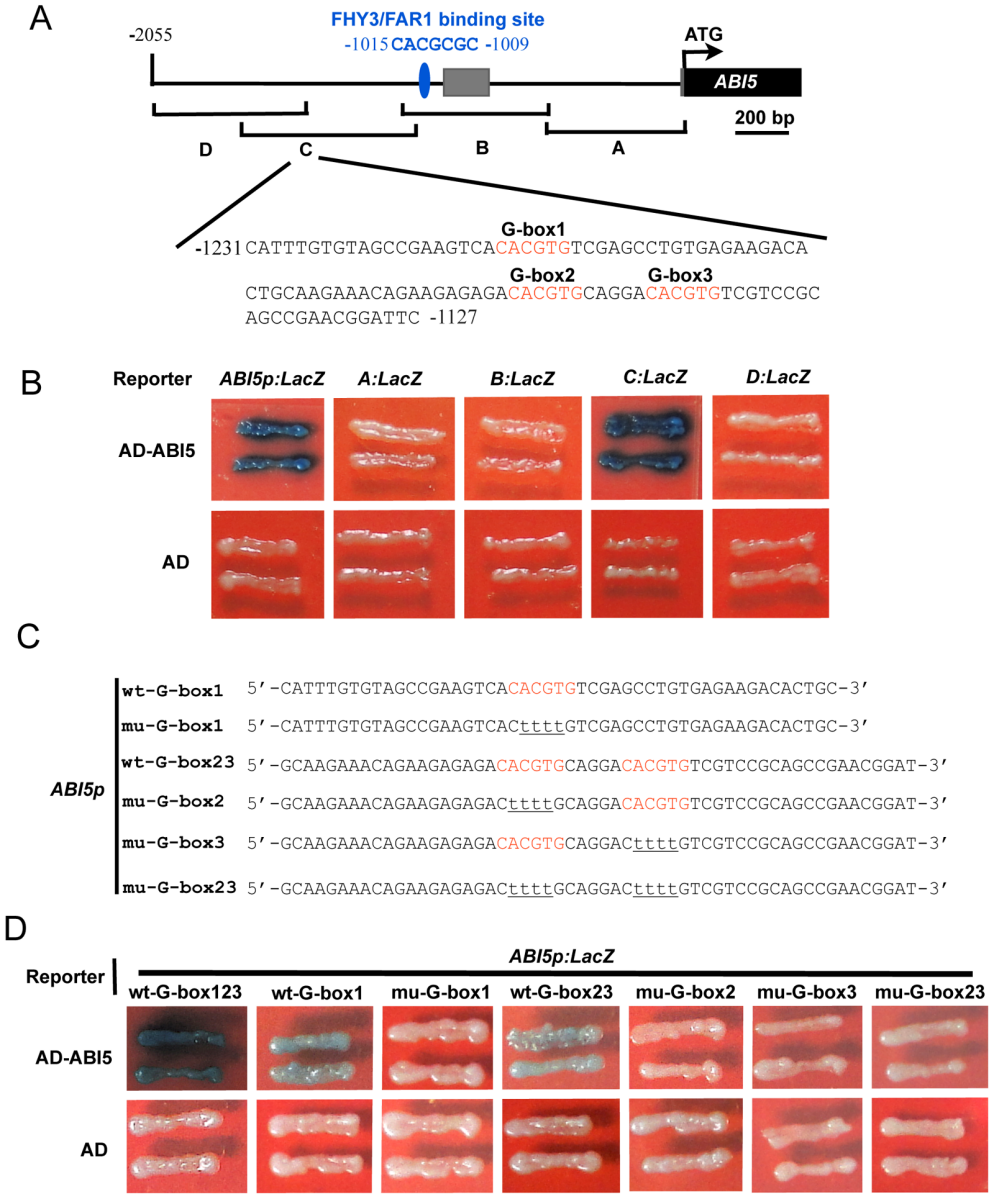
ABI5 directly binds to its own promoter. (A) Diagram of the promoter fragments of the *ABI5* promoter and the sequence of a subfragment containing three G-box motifs in the C fragment. The adenine residue of the translational start codon (ATG) was assigned position +1, and the numbers flanking the sequences of the subfragment were counted based on this number. A, B, C, and D indicate the corresponding promoter fragments used in yeast one-hybrid assays shown in (B). (B) Yeast one hybrid assays showing that ABI5 binds to the C fragments of its own promoter. Empty vector expressing AD domain alone was used as negative controls. (C) Diagram of the wild type (wt) and mutant (mu) *ABI5* subfragments used to drive *Lac*Z reporter gene expression in yeast one-hybrid assays. Wild type G-box elements are shown in red, and nucleotide substitutions in the mutant fragments are underlined. (D) Yeast one-hybrid assays showing that all three G-box motifs mediate ABI5 binding to its promoter. The subfragment of the *ABI5* promoter was mutated to abolish G-box1, 2, 3 alone, or both G-box2 and 3, and used to drive *Lac*Z reporter gene expression. In these assays, the respective CACGTG was mutated to CttttG to facilitate mutagenesis reactions.

We performed yeast one-hybrid assays to test whether other transcription factors may bind to the 2-kb promoter fragment of *ABI5*. Unexpectedly, it was shown that ABI5 itself binds directly to its promoter (Figure 8A and 8B), suggesting that ABI5 may play a role in regulating its own expression. To further confirm this conclusion, we divided the *ABI5* promoter into four overlapping fragments, designated A, B, C, and D, and generated yeast one-hybrid reporter constructs allowing the respective fragments to drive *Lac*Z reporter gene expression (Figure 8A). Interestingly, ABI5 binds only to the C fragment of the *ABI5* promoter (Figure 8B), indicating that the C fragment contains the ABI5 binding sites.

Because ABI5 binds to the class of G-box (CACGTG) motifs known as ABA response elements (ABREs) [37], we then analyzed the distribution of G-box motifs in the C fragment of the *ABI5* promoter. Our analysis showed that there are three typical G-box motifs present in the C fragment, which are located ∼200 to 300 bp upstream of the transcription start site and are very close to each other (Figure 8A). To delineate the exact G-box motifs in the *ABI5* promoter that are bound by ABI5, we generated several reporter constructs to allow the subfragment 1 (which contains only G-box 1) or subfragment 2 (which contains both G-box 2 and 3) to drive *Lac*Z reporter gene expression, respectively, in yeast cells (Figure 8C). Our results show that for subfragment 1, ABI5 binds to the wild type, but not the mutant (in which CACGTG was mutated to CttttG) fragment, whereas for subfragment 2, mutations of G-box 2 or 3 alone or together all abolished ABI5 binding (Figure 8D). Together, our data demonstrate that all three G-box motifs located in the C fragment mediate ABI5 binding to the *ABI5* promoter.

To further explore the biological significance of ABI5 binding to its own promoter, we employed again the transient transfection system using *Arabidopsis* protoplasts. As shown in Figure 7B, ABI5 alone also activates its own promoter, although not as robustly as HY5. Interestingly, when BBX21 was co-expressed with *ABI5*, BBX21 was also able to decrease ABI5-activated ABI5 expression (Figure 7B). In addition, when BBX21, ABI5 and HY5 were expressed together, the reporter gene expression was much lower than when ABI5 and HY5 were co-expressed (Figure 7B). These data indicate that BBX21 negatively regulates both HY5- and ABI5-activated *ABI5* expression.

**Figure 9:**
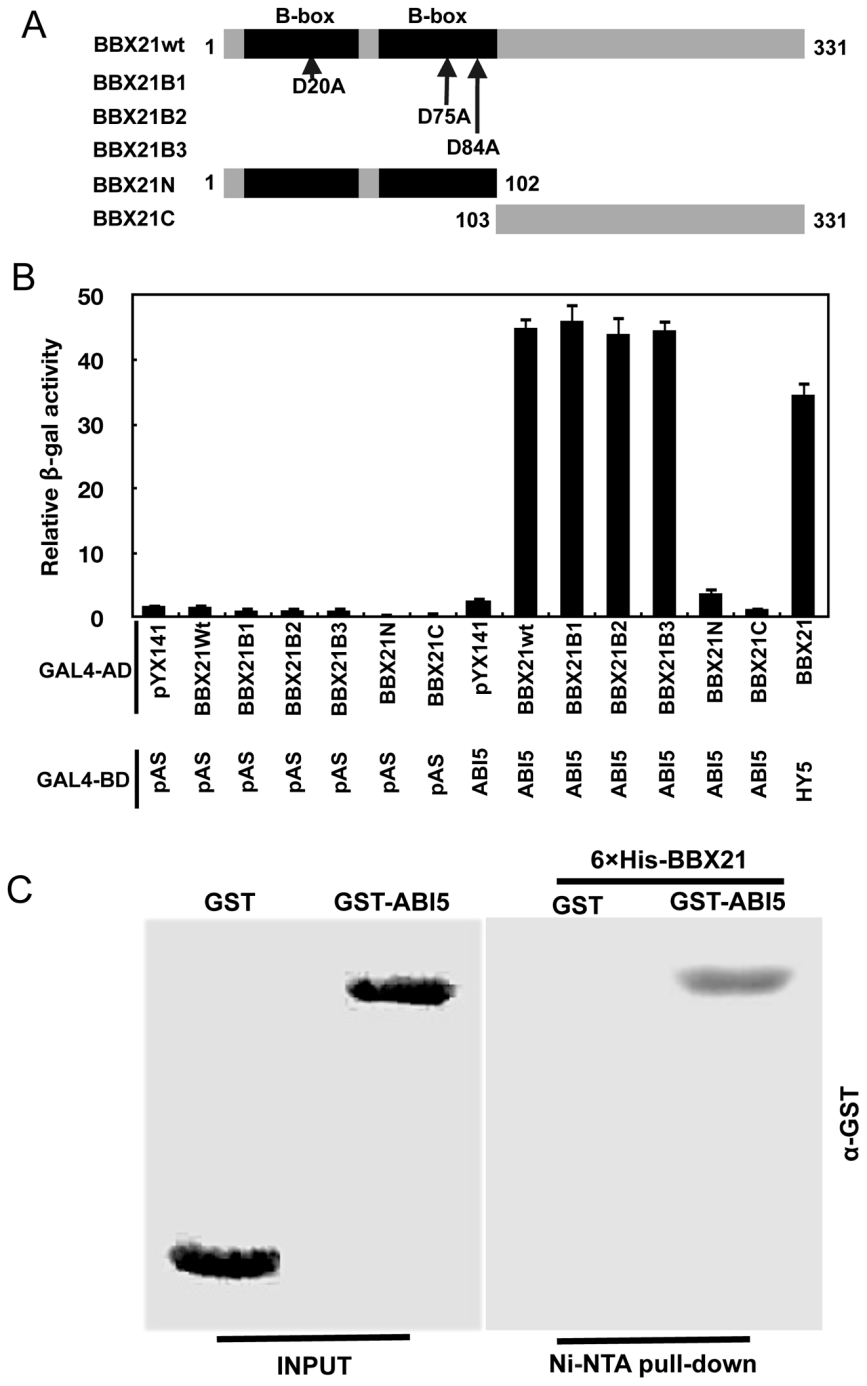
BBX21 physically interacts with ABI5. (**A**) **Schematic representation of the domain structures of BBX21 showing mutations in the B-box domain.** (B) Yeast two-hybrid interactions between BBX21 and ABI5 proteins. Error bars indicate SE (n = 3). (C) In vitro pull down of ABI5 with BBX21. The GST-ABI5 proteins pulled down with 6×His-BBX21 were detected by anti-GST antibody. Input, 5% of the purified GST-tagged target proteins used in pull-down assays.

**Figure 10:**
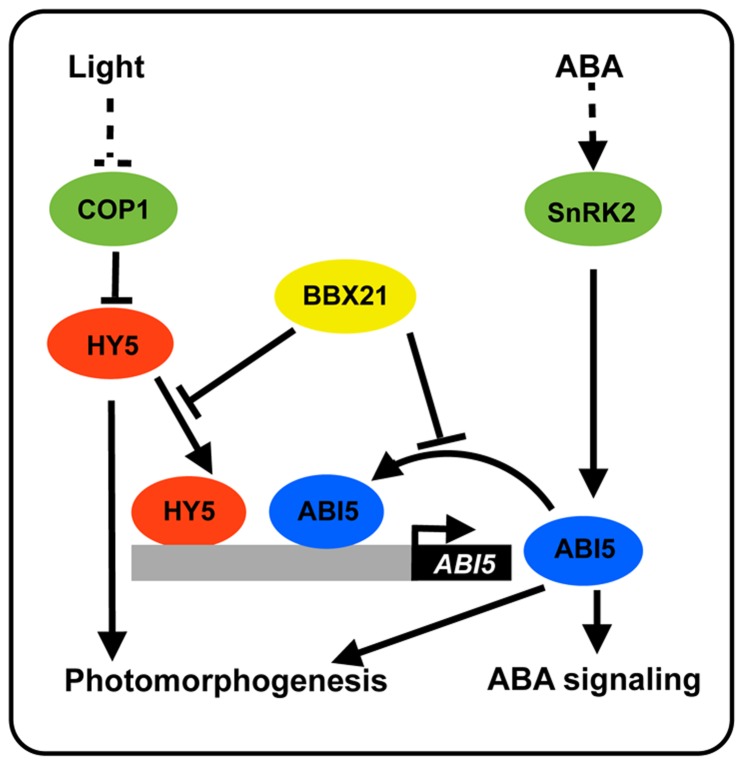
A working model depicting how BBX21 works in concert with HY5 and ABI5 on the *ABI5* promoter to integrate light and ABA signaling. HY5 acts as a transcriptional activator of *ABI5* expression [33]. ABI5 directly binds to its own promoter through three typical G-box motifs, and activates the expression of itself. BBX21 acts as a negative regulator of *ABI5* expression, possibly by interfering with the binding of HY5 and ABI5 to the *ABI5* promoter. ABI5 may play a positive role in photomorphogenesis [33].

It seems likely that BBX21 may act through a common mechanism (i.e. sequestration of transcription factors) to regulate the transcriptional activities of both HY5 and ABI5. To confirm this hypothesis, we performed yeast two-hybrid and in vitro pull-down assays to investigate whether there is a direct physical interaction between BBX21 and ABI5. Results from these experiments showed that BBX21 indeed interacts directly with ABI5 (Figure 9A, 9B and 9C). Interestingly, individual substitutions of three conserved Asp residues in the B-box domain of BBX21 (i.e. D20A, D75A and D84A), which all abolished the direct interaction between BBX21 and HY5 due to presumable disruption of the structure of the B-box domain [15], did not affect the physical interaction between BBX21 and ABI5 (Figure 9A and 9B). These results suggest that the B-box domain may not mediate the interaction between BBX21 and ABI5. To further investigate which region of BBX21 may be responsible for its interaction with ABI5, we generated the yeast two-hybrid constructs in which the N- (the B-box domain) or C-terminal domain of BBX21 was fused, respectively, with GAL4 activation domain (GAL4-AD). As shown in Figure 9B, our results show that either domain was not able to interact with ABI5 in yeast cells (the interaction of BBX21 and HY5 was included as the positive control), implying that the entire protein of BBX21 is required for interacting with ABI5. Taken together, our data indicate that HY5 and ABI5 are both direct activators of *ABI5* expression, whereas BBX21 acts as a negative regulator, possibly by interacting with both transcription factors and interfering with their binding to the *ABI5* promoter (Figure 10).

## Discussion

Light and ABA are the external environmental and the endogenous hormonal cues, respectively, that both play important roles in the control of seed germination and seedling development. The ability of plants to integrate external signals with internal regulatory pathways is vital for their survival [38]–[39]. However, the molecular mechanisms underlying the cross-talk between light and ABA are just starting to be understood. It was reported that HY5, a well-studied transcription factor involved in promoting seedling photomorphogenesis, mediates ABA responses in seed germination, early seedling growth, and root development in *Arabidopsis* by directly binding to the *ABI5* promoter and activating its expression [33]. A recent study reported that FAR-RED ELONGATED HYPOCOTYL3 (FHY3) and FAR-RED IMPAIRED RESPONSE1 (FAR1), two key transcription factors in the phytochrome A pathway, are positive regulators of ABA signaling by directly activating *ABI5* expression [40]. In addition, it was shown that *ABI5* is also a direct target of PIF1 (also known as PIL5), a phytochrome-interacting bHLH transcription factor [41]. Therefore, it is evident that multiple transcription factors in light signaling pathway may regulate ABA responses by regulating *ABI5* expression. In this study, we show that BBX21, another transcriptional regulator implicated in regulating seedling photomorphogenesis, negatively regulates *ABI5* expression by interfering with HY5 binding to the *ABI5* promoter. Interestingly, ABI5 was also shown to directly activate its own expression, whereas BBX21 negatively regulates this activation by directly interacting with *ABI5*. Therefore, BBX21 acts as a transcriptional repressor of ABI5, possibly by modulating the binding activities of both HY5 and ABI5 to the *ABI5* promoter (Figure 10).

On the other hand, several components of ABA signaling pathway have been shown to be implicated in photomorphogenesis. For example, overexpression of *ABI5* in wild type background led to shorter hypocotyls of the transgenic seedlings in FR, R and B light conditions [33]. Consistent with this observation, disruption of RPN10 and KEG, which stabilize the ABI5 protein, both caused decreased hypocotyl growth of the mutants [42]–[43]. Another study proposed that seeds in shallow soil could sense their light environment through phytochrome B, so as to down-regulate *ABI3* expression and promote hypocotyl growth [44]. In darkness, the hypocotyl length was slightly longer in *abi3* mutants but shorter in *ABI3* overexpression plants [44]. Therefore, both ABI3 and ABI5 may play positive roles in photomorphogenesis, and ABA seems to inhibit hypocotyl elongation of seedlings, which may confer better adaptation of seedlings to environmental stresses.

It is interesting that several families of transcription factors directly bind to the *ABI5* promoter and regulate its expression. FHY3/FAR1 bind to the FHY3/FAR1-binding site (FBS; CACGCGC) [40], whereas ABI5 binds to the G-box motifs which are ∼130 bp upstream of FBS in the *ABI5* promoter (Figure 8). In addition, HY5 presumably binds to a typical G-box motif in the *ABI5* promoter [33] which is ∼500 bp upstream of the ABI5-binding sites. Although both HY5 and ABI5 belong to the bZIP transcription factor family and preferentially bind to G-box motif, they apparently bind to different G-box motifs in the *ABI5* promoter, suggesting that additional sequences around the core G-box motif may be important for selective binding of these bZIP transcription factors. The binding site of PIF1 (which also binds to G-box motif) in the *ABI5* promoter remains to be determined. Moreover, ABI4, an AP2/ERF family transcription factor, was shown to bind to CE1-like element (CACCG) in the 5′-untranslated region of *ABI5* [45]. Therefore, at least FHY3/FAR1, ABI5, HY5 and ABI4 directly bind to the *ABI5* promoter, and they seem to occupy different regions of the *ABI5* promoter. Interestingly, based on our data and those reported in previous studies, all of these transcription factors function as activators of *ABI5*. How their activities are coordinated and whether they could regulate each other on the *ABI5* promoter remain to be elucidated. Since BBX21 is the only negative regulator identified so far on the *ABI5* promoter, it remains unknown whether BBX21 could also regulate the binding activities of other transcription factors, such as FHY3/FAR1 and ABI4. In addition, it was shown that ABI3, a B3-domain containing transcription factor, functions as essential regulator upstream of *ABI5* [31]. *ABI5* expression is greatly reduced in the *abi3* mutants, and *35S:ABI5* could rescue ABA insensitivity of *abi3* [31]. Whether ABI3 is also a direct regulator of *ABI5* awaits further investigation. Together, the *ABI5* promoter may represent a converging point on which the transcriptional regulators of light and ABA signaling pathways integrate the signals of both pathways by fine-tuning *ABI5* expression.

In addition, it was documented that light could affect ABA metabolism in germinating seeds, and that PIF1 represses seed germination by regulating the expression of ABA biosynthetic and catabolic genes [46]–[48]. Moreover, PIF1 interacts with ABI3 to activate the expression of *SOMNUS (SOM)* in imbibed seeds [49], suggesting that light and ABA signals also converge on other gene promoters. Together, it is evident that light and ABA integrate at multiple layers to fine-tune seed germination and seedling development, which may ensure the young seedlings to better adapt to their environmental stresses and dynamic light conditions.

## Materials and Methods

### Plant materials and growth conditions

The *bbx21-1* [15], *hy5-215* [6], *aba1-5* [50] and *abi4-101* [36] mutants are of the Columbia (Col) ecotype, and the *bbx21-2* [15], *aba1-1* [51], *abi2-1* [34] and *abi3-1* [35] mutants are of the Landsberg (L*er*) ecotype, and the *abi5-1* [27] mutant is of the Wassilewskija (Ws) ecotype and have been described previously. Seeds were surface-sterilized with 30% commercial Clorox bleach and 0.02% Triton X-100 for 15 min and washed three times with sterile water, then plated on GM medium supplemented with 0.8% Bacto-agar (Difco) and 1% sucrose. The plates were kept at 4°C for 3 days for stratification and then transferred to light chambers maintained at 22°C.

### Analyses of ABA responses and quantification of endogenous ABA levels

For root growth measurement shown in Figures 1A and 1B, all seedlings were grown vertically on GM plates with or without 1 µM ABA (Sigma-Aldrich) or 100 mM NaCl for 7 d after stratification under long-day conditions (16 h light/8 h dark).

For the germination assay, at least 150 seeds for each genotype were sterilized and plated on MS medium supplemented with or without various concentration of ABA or NaCl. Germination was defined as the first sign of radicle tip emergence and scored daily until the 7th day of the incubation, and the germination results were calculated based on at least three independent experiments.

Stomatal closing assays were conducted as previously described [52]. Rosette leaves were floated in KCl-Tris solution (50 mM KCl, 10 mM Mes-Tris, pH 6.15), and exposed to light (150 µmol m^−2^ s^−1^) for 3 h. Subsequently, rosette leaves were incubated in KCl-Tris solution with or without 0.5 µM ABA assay under the light condition for an additional 3 h.

Water loss was measured in ∼1 g detached rosette leaves from 3-week-old wild type and mutant plants grown in long-day condition. The leaves were weighed immediately after detaching, kept on the laboratory bench and weighted at the indicated times. Water loss shows the percentage of weight loss at the indicated time versus initial fresh weight. To minimize variation, three independent experiments were performed, and similar results were obtained.

Endogenous ABA was quantified in germinated seeds by liquid chromatography coupled to electrospray tandem mass spectrometry as described previously [53].

### Measurement of hypocotyl length

To measure the hypocotyl length of seedlings, seeds were cold-treated at 4°C for 3 days and then transferred to continuous white light for 8 h to induce uniform germination. Then, the seeds were transferred to monochromatic light conditions (B, R, and FR light) and incubated at 22°C for 4 d. The hypocotyl length of seedlings was measured using ImageJ software.

### Real-Time qRT-PCR

Total RNA was extracted from Arabidopsis seedlings using the RNeasy plant mini kit (Qiagen). Then, cDNAs were synthesized from 2 µg total RNA using SuperScript II first-strand cDNA synthesis system (Invitrogen) according to the manufacturer's instructions. Quantitative PCR was performed using the CFX96 real-time PCR detection system (Bio-Rad) and SYBR Green PCR Master Mix (Applied Biosystems). PCR reactions were performed in triplicate for each sample, and the expression levels were normalized to that of a ubiquitin gene. All primers used for this assay are listed in Supplemental Table S1.

### EMSA

EMSAs were performed using biotin-labeled probes and the Light Shift Chemiluminescent EMSA kit (Pierce) as described previously [54]. Briefly, 0.5 µg of GST or GST fusion proteins were incubated together with biotin-labeled probes in 20 µl reaction mixtures containing 10 mM Tris-HCl, 150 mM KCl, 1 mM DTT, 50 ng/ml poly (dI-dC), 2.5% glycerol, 0.05% Nonidet P-40, 100 mM ZnCl2, and 0.5 µg/ml BSA for 20 min at room temperature and separated on 6% native polyacrylamide gels in Tris-glycine buffer. The labeled probes were detected according to the instructions provided with the EMSA kit. The sequences of the complementary oligonucleotides used to generate the biotin-labeled probes are shown in Supplemental Table S1.

### Protoplast assay

*Arabidopsis* mesophyll cell protoplasts were prepared and transfected as described previously [55]. The promoter-reporter used was the 2-kb *ABI5* promoter driving firefly luciferase *(pPCV814-ABI5p-LUC)*. The full-length HY5, BBX21, and ABI5 driven by the cauliflower mosaic virus 35S promoter *(pRLT2-HY5, pRTL2-BBX21* and *pRTL2-ABI5)* were used as the effectors. The dual luciferase kit (Promega) was used for detection of reporter activity. Renilla luciferase driven by a full-length cauliflower mosaic virus 35S promoter (pRNL) was used as an internal control.

### Yeast assays

Yeast assays were performed as previously described [54]. Transformants were grown on proper drop-out plates containing X-gal (5-bromo-4-chloro-3-indolyl-β-D-galactopyranoside) for blue color development. Yeast transformation and liquid assay were conducted as described in the Yeast Protocols Handbook (Clontech).

### In vitro pull-down assay

For in vitro pull-down assays, 2 µg purified 6×His-BBX21 proteins were bound to Ni-NTA magnetic beads (Qiagen) for 2 hours at 4°C, followed by overnight incubation at 4°C with 2 µg purified GST or GST-ABI5 proteins, then washed by washing buffer (10 mM Tris pH 8.0, 100 mM NaCl, and 0.1% Triton X-100, and 5 mM imidazol). Pulled-down proteins mixed with Ni-NTA magnetic beads was spun down and boiled for 10 minutes prior to being loaded to 12% SDS polyacrylamide gels and were detected by Western blot using anti-GST antibody (Sigma-Aldrich).

## Supporting Information

Figure S1 ABA levels in the dry or germinating seeds of the wild type and *bbx21* mutants. Data are means of three independent experiments, and error bars represent SD.
(TIF)

Figure S2 Germination rates of three *hy5* mutants (*hy5-215*, *hy5-ks50* and *hy5-1*) and their corresponding wild type controls under mock (A), 1 µM (B), 3 µM (C) and 5 µM (D) ABA treatments.
(TIF)

Figure S3 The expression levels of *ABI1* (A), *ABI2* (B), *ABI3* (C), *ABI4* (D) and *ABI5* (E) in 2-d-old germinating seeds of *bbx21-1* (Col) and *bbx21-2* (L*er*) mutants and their corresponding wild type controls treated with mock or 0.5 µM ABA. Data are means of three independent experiments, and error bars represent SD.
(TIF)

Table S1 A list of primers used in this study.
(DOC)

## References

[1] Rizzini L, Favory JJ, Cloix C, Faggionato D, O'Hara A, et al (2011) Perception of UV-B by the *Arabidopsis* UVR8 protein. Science 332: 103–106. 21454788 10.1126/science.1200660

[2] Yi C, Deng XW (2005) COP1-from plant photomorphogenesis to mammalian tumorigenesis. Trends Cell Biol 15: 618–625. 16198569 10.1016/j.tcb.2005.09.007

[3] Osterlund MT, Hardtke CS, Wei N, Deng XW (2000) Targeted destabilization of HY5 during light-regulated development of *Arabidopsis* . Nature 405: 462–466. 10839542 10.1038/35013076

[4] Holm M, Ma LG, Qu LJ, Deng XW (2002) Two interacting bZIP proteins are direct targets of COP1-mediated control of light-dependent gene expression in *Arabidopsis* . Genes Dev 16: 1247–1259. 12023303 10.1101/gad.969702 PMC186273

[5] Koornneef M, Rolff E, Spruit CJP (1980) Genetic control of light-inhibited hypocotyl elongation in *Arabidopsis thaliana* (L.). Heynh Z Pflanzenphysiol 100: 147–160.

[6] Oyama T, Shimura Y, Okada K (1997) The *Arabidopsis HY5* gene encodes a bZIP protein that regulates stimulus-induced development of root and hypocotyl. Genes Dev 11: 2983–2995. 9367981 10.1101/gad.11.22.2983 PMC316701

[7] Ulm R, Baumann A, Oravecz A, Mate Z, Adam E, et al (2004) Genome-wide analysis of gene expression reveals function of the bZIP transcription factor HY5 in the UV-B response of *Arabidopsis* . Proc Natl Acad Sci U S A 101: 1397–1402. 14739338 10.1073/pnas.0308044100 PMC337064

[8] Lee J, He K, Stolc V, Lee H, Figueroa P, et al (2007) Analysis of transcription factor HY5 genomic binding sites revealed its hierarchical role in light regulation of development. Plant Cell 19: 731–749. 17337630 10.1105/tpc.106.047688 PMC1867377

[9] Zhang H, He H, Wang X, Wang X, Yang X, et al (2011) Genome-wide mapping of the HY5-mediated gene networks in *Arabidopsis* that involve both transcriptional and post-transcriptional regulation. Plant J 65: 346–358. 21265889 10.1111/j.1365-313X.2010.04426.x

[10] Khanna R, Kronmiller B, Maszle DR, Coupland G, Holm M, et al (2009) The *Arabidopsis* B-box zinc finger family. Plant Cell 21: 3416–3420. 19920209 10.1105/tpc.109.069088 PMC2798317

[11] Putterill J, Robson F, Lee K, Simon R, Coupland G (1995) The *CONSTANS* gene of *Arabidopsis* promotes flowering and encodes a protein showing similarities to zinc finger transcription factors. Cell 80: 847–857. 7697715 10.1016/0092-8674(95)90288-0

[12] Sarmiento F (2013) The BBX subfamily IV: Additional cogs and sprockets to fine-tune light-dependent development. Plant Signal Behav 8: e23831. 23425851 10.4161/psb.23831 PMC7030190

[13] Chang CS, Maloof JN, Wu SH (2011) COP1-mediated degradation of BBX22/LZF1 optimizes seedling development in Arabidopsis. Plant Physiol 156: 228–239. 21427283 10.1104/pp.111.175042 PMC3091042

[14] Chang CS, Li YH, Chen LT, Chen WC, Hsieh WP, et al (2008) LZF1, a HY5-regulated transcriptional factor, functions in *Arabidopsis* de-etiolation. Plant J 54: 205–219. 18182030 10.1111/j.1365-313X.2008.03401.x

[15] Datta S, Hettiarachchi C, Johansson H, Holm M (2007) SALT TOLERANCE HOMOLOG2, a B-box protein in *Arabidopsis* that activates transcription and positively regulates light-mediated development. Plant Cell 19: 3242–3255. 17965270 10.1105/tpc.107.054791 PMC2174709

[16] Datta S, Johansson H, Hettiarachchi C, Irigoyen ML, Desai M, et al (2008) LZF1/SALT TOLERANCE HOMOLOG3, an *Arabidopsis* B-box protein involved in light-dependent development and gene expression, undergoes COP1-mediated ubiquitination. Plant Cell 20: 2324–2338. 18796637 10.1105/tpc.108.061747 PMC2570732

[17] Gangappa SN, Crocco CD, Johansson H, Datta S, Hettiarachchi C, et al (2013) The *Arabidopsis* B-BOX protein BBX25 interacts with HY5, negatively regulating BBX22 expression to suppress seedling photomorphogenesis. Plant Cell 25: 1243–1257. 23624715 10.1105/tpc.113.109751 PMC3663265

[18] Holm M, Hardtke CS, Gaudet R, Deng XW (2001) Identification of a structural motif that confers specific interaction with the WD40 repeat domain of *Arabidopsis* COP1. EMBO J 20: 118–127. 11226162 10.1093/emboj/20.1.118 PMC140188

[19] Indorf M, Cordero J, Neuhaus G, Rodriguez-Franco M (2007) Salt tolerance (STO), a stress-related protein, has a major role in light signalling. Plant J 51: 563–574. 17605755 10.1111/j.1365-313X.2007.03162.x

[20] Jiang L, Wang Y, Li QF, Bjorn LO, He JX, et al (2012) *Arabidopsis* STO/BBX24 negatively regulates UV-B signaling by interacting with COP1 and repressing HY5 transcriptional activity. Cell Res 22: 1046–1057. 22410790 10.1038/cr.2012.34 PMC3367526

[21] Kumagai T, Ito S, Nakamichi N, Niwa Y, Murakami M, et al (2008) The common function of a novel subfamily of B-Box zinc finger proteins with reference to circadian-associated events in *Arabidopsis thaliana* . Biosci Biotechnol Biochem 72: 1539–1549. 18540109 10.1271/bbb.80041

[22] Yan H, Marquardt K, Indorf M, Jutt D, Kircher S, et al (2011) Nuclear localization and interaction with COP1 are required for STO/BBX24 function during photomorphogenesis. Plant Physiol 156: 1772–1782. 21685177 10.1104/pp.111.180208 PMC3149933

[23] Cutler SR, Rodriguez PL, Finkelstein RR, Abrams SR (2010) Abscisic acid: emergence of a core signaling network. Annu Rev Plant Biol 61: 651–679. 20192755 10.1146/annurev-arplant-042809-112122

[24] Nakashima K, Yamaguchi-Shinozaki K (2013) ABA signaling in stress-response and seed development. Plant Cell Rep 32: 959–970. 23535869 10.1007/s00299-013-1418-1

[25] Kobayashi Y, Murata M, Minami H, Yamamoto S, Kagaya Y, et al (2005) Abscisic acid-activated SNRK2 protein kinases function in the gene-regulation pathway of ABA signal transduction by phosphorylating ABA response element-binding factors. Plant J 44: 939–949. 16359387 10.1111/j.1365-313X.2005.02583.x

[26] Umezawa T, Sugiyama N, Mizoguchi M, Hayashi S, Myouga F, et al (2009) Type 2C protein phosphatases directly regulate abscisic acid-activated protein kinases in *Arabidopsis* . Proc Natl Acad Sci U S A 106: 17588–17593. 19805022 10.1073/pnas.0907095106 PMC2754379

[27] Finkelstein RR, Lynch TJ (2000) The *Arabidopsis* abscisic acid response gene *ABI5* encodes a basic leucine zipper transcription factor. Plant Cell 12: 599–609. 10760247 10.1105/tpc.12.4.599 PMC139856

[28] Finkelstein RR, Wang ML, Lynch TJ, Rao S, Goodman HM (1998) The *Arabidopsis* abscisic acid response locus *ABI4* encodes an APETALA 2 domain protein. Plant Cell 10: 1043–1054. 9634591 10.1105/tpc.10.6.1043 PMC144030

[29] Parcy F, Valon C, Raynal M, Gaubier-Comella P, Delseny M, et al (1994) Regulation of gene expression programs during *Arabidopsis* seed development: roles of the *ABI3* locus and of endogenous abscisic acid. Plant Cell 6: 1567–1582. 7827492 10.1105/tpc.6.11.1567 PMC160544

[30] Lopez-Molina L, Mongrand S, Chua NH (2001) A postgermination developmental arrest checkpoint is mediated by abscisic acid and requires the ABI5 transcription factor in Arabidopsis. Proc Natl Acad Sci U S A 98: 4782–4787. 11287670 10.1073/pnas.081594298 PMC31911

[31] Lopez-Molina L, Mongrand S, McLachlin DT, Chait BT, Chua NH (2002) ABI5 acts downstream of ABI3 to execute an ABA-dependent growth arrest during germination. Plant J 32: 317–328. 12410810 10.1046/j.1365-313x.2002.01430.x

[32] Piskurewicz U, Jikumaru Y, Kinoshita N, Nambara E, Kamiya Y, et al (2008) The gibberellic acid signaling repressor RGL2 inhibits *Arabidopsis* seed germination by stimulating abscisic acid synthesis and ABI5 activity. Plant Cell 20: 2729–2745. 18941053 10.1105/tpc.108.061515 PMC2590721

[33] Chen H, Zhang J, Neff MM, Hong SW, Zhang H, et al (2008) Integration of light and abscisic acid signaling during seed germination and early seedling development. Proc Natl Acad Sci U S A 105: 4495–4500. 18332440 10.1073/pnas.0710778105 PMC2393781

[34] Leung J, Merlot S, Giraudat J (1997) The *Arabidopsis ABSCISIC ACID-INSENSITIVE2 (ABI2)* and *ABI1* genes encode homologous protein phosphatases 2C involved in abscisic acid signal transduction. Plant Cell 9: 759–771. 9165752 10.1105/tpc.9.5.759 PMC156954

[35] Parcy F, Valon C, Kohara A, Misera S, Giraudat J (1997) The *ABSCISIC ACID-INSENSITIVE3, FUSCA3*, and *LEAFY COTYLEDON1* loci act in concert to control multiple aspects of *Arabidopsis* seed development. Plant Cell 9: 1265–1277. 9286105 10.1105/tpc.9.8.1265 PMC156996

[36] Laby RJ, Kincaid MS, Kim D, Gibson SI (2000) The *Arabidopsis* sugar-insensitive mutants *sis4 *and *sis5 are defective in abscisic acid synthesis and response.* . Plant J 23: 587–596. 10972885 10.1046/j.1365-313x.2000.00833.x

[37] Reeves WM, Lynch TJ, Mobin R, Finkelstein RR (2011) Direct targets of the transcription factors ABA-Insensitive(ABI)4 and ABI5 reveal synergistic action by ABI4 and several bZIP ABA response factors. Plant Mol Biol 75: 347–363. 21243515 10.1007/s11103-011-9733-9 PMC3044226

[38] Achard P, Cheng H, De Grauwe L, Decat J, Schoutteten H, et al (2006) Integration of plant responses to environmentally activated phytohormonal signals. Science 311: 91–94. 16400150 10.1126/science.1118642

[39] Casal JJ, Fankhauser C, Coupland G, Blazquez MA (2004) Signalling for developmental plasticity. Trends Plant Sci 9: 309–314. 15165563 10.1016/j.tplants.2004.04.007

[40] Tang W, Ji Q, Huang Y, Jiang Z, Bao M, et al (2013) FAR-RED ELONGATED HYPOCOTYL3 and FAR-RED IMPAIRED RESPONSE1 transcription factors integrate light and abscisic acid signaling in *Arabidopsis* . Plant Physiol 163: 857–866. 23946351 10.1104/pp.113.224386 PMC3793063

[41] Oh E, Kang H, Yamaguchi S, Park J, Lee D, et al (2009) Genome-wide analysis of genes targeted by PHYTOCHROME INTERACTING FACTOR 3-LIKE5 during seed germination in *Arabidopsis* . Plant Cell 21: 403–419. 19244139 10.1105/tpc.108.064691 PMC2660632

[42] Smalle J, Kurepa J, Yang P, Emborg TJ, Babiychuk E, et al (2003) The pleiotropic role of the 26S proteasome subunit RPN10 in *Arabidopsis* growth and development supports a substrate-specific function in abscisic acid signaling. Plant Cell 15: 965–980. 12671091 10.1105/tpc.009217 PMC152342

[43] Stone SL, Williams LA, Farmer LM, Vierstra RD, Callis J (2006) KEEP ON GOING, a RING E3 ligase essential for *Arabidopsis* growth and development, is involved in abscisic acid signaling. Plant Cell 18: 3415–3428. 17194765 10.1105/tpc.106.046532 PMC1785414

[44] Mazzella MA, Arana MV, Staneloni RJ, Perelman S, Rodriguez Batiller MJ, et al (2005) Phytochrome control of the *Arabidopsis* transcriptome anticipates seedling exposure to light. Plant Cell 17: 2507–2516. 16024587 10.1105/tpc.105.034322 PMC1197430

[45] Bossi F, Cordoba E, Dupré P, Mendoza MS, Román CS, et al (2009) The *Arabidopsis* ABA-INSENSITIVE (ABI) 4 factor acts as a central transcription activator of the expression of its own gene, and for the induction of ABI5 and SBE2.2 genes during sugar signaling. Plant J 59: 359–374. 19392689 10.1111/j.1365-313X.2009.03877.x

[46] Oh E, Kim J, Park E, Kim JI, Kang C, et al (2004) PIL5, a phytochrome-interacting basic helix-loop-helix protein, is a key negative regulator of seed germination in *Arabidopsis thaliana* . Plant Cell 16: 3045–3058. 15486102 10.1105/tpc.104.025163 PMC527197

[47] Oh E, Yamaguchi S, Hu J, Yusuke J, Jung B, et al (2007) PIL5, a phytochrome-interacting bHLH protein, regulates gibberellin responsiveness by binding directly to the *GAI *and *RGA* promoters in *Arabidopsis* seeds. Plant Cell 19: 1192–1208. 17449805 10.1105/tpc.107.050153 PMC1913757

[48] Seo M, Hanada A, Kuwahara A, Endo A, Okamoto M, et al (2006) Regulation of hormone metabolism in *Arabidopsis* seeds: phytochrome regulation of abscisic acid metabolism and abscisic acid regulation of gibberellin metabolism. Plant J 48: 354–366. 17010113 10.1111/j.1365-313X.2006.02881.x

[49] Park J, Lee N, Kim W, Lim S, Choi G (2011) ABI3 and PIL5 collaboratively activate the expression of *SOMNUS* by directly binding to its promoter in imbibed *Arabidopsis* seeds. Plant Cell 23: 1404–1415. 21467583 10.1105/tpc.110.080721 PMC3101561

[50] Leon-Kloosterziel KM, Gil MA, Ruijs GJ, Jacobsen SE, Olszewski NE, et al (1996) Isolation and characterization of abscisic acid-deficient *Arabidopsis* mutants at two new loci. Plant J 10: 655–661. 8893542 10.1046/j.1365-313x.1996.10040655.x

[51] Koornneef JM, Brinkhorst-Van der Swan DLC, Karssen CM (1982) The isolation of abscisic acid (ABA)-deficient mutants by selection of induced revertants in non-germinating gibberellin-sensitive lines of *Arabidopsis thaliana* . Theor Appl Genet 61: 385–393. 24270501 10.1007/BF00272861

[52] Ren X, Chen Z, Liu Y, Zhang H, Zhang M, et al (2010) ABO3, a WRKY transcription factor, mediates plant responses to abscisic acid and drought tolerance in *Arabidopsis* . Plant J 63: 417–429. 20487379 10.1111/j.1365-313X.2010.04248.x PMC3117930

[53] Pan X, Welti R, Wang X (2010) Quantitative analysis of major plant hormones in crude plant extracts by high-performance liquid chromatography-mass spectrometry. Nat Protoc 5: 986–992. 20448544 10.1038/nprot.2010.37

[54] Li J, Li G, Gao S, Martinez C, He G, et al (2010) Plant Cell 22: 3634–3649. 21097709 10.1105/tpc.110.075788 PMC3015127

[55] Yoo SD, Cho YH, Sheen J (2007) *Arabidopsis* mesophyll protoplasts: a versatile cell system for transient gene expression analysis. Nat Protoc 2: 1565–1572. 17585298 10.1038/nprot.2007.199

