# Supplementary figures and images for: Convergence of Light and ABA Signaling on the ABI5 Promoter
Source: PLoS Genet. 2014 Feb 27;10(2):e1004197. doi: 10.1371/journal.pgen.1004197 (PMC3937224; doi:10.1371/journal.pgen.1004197)

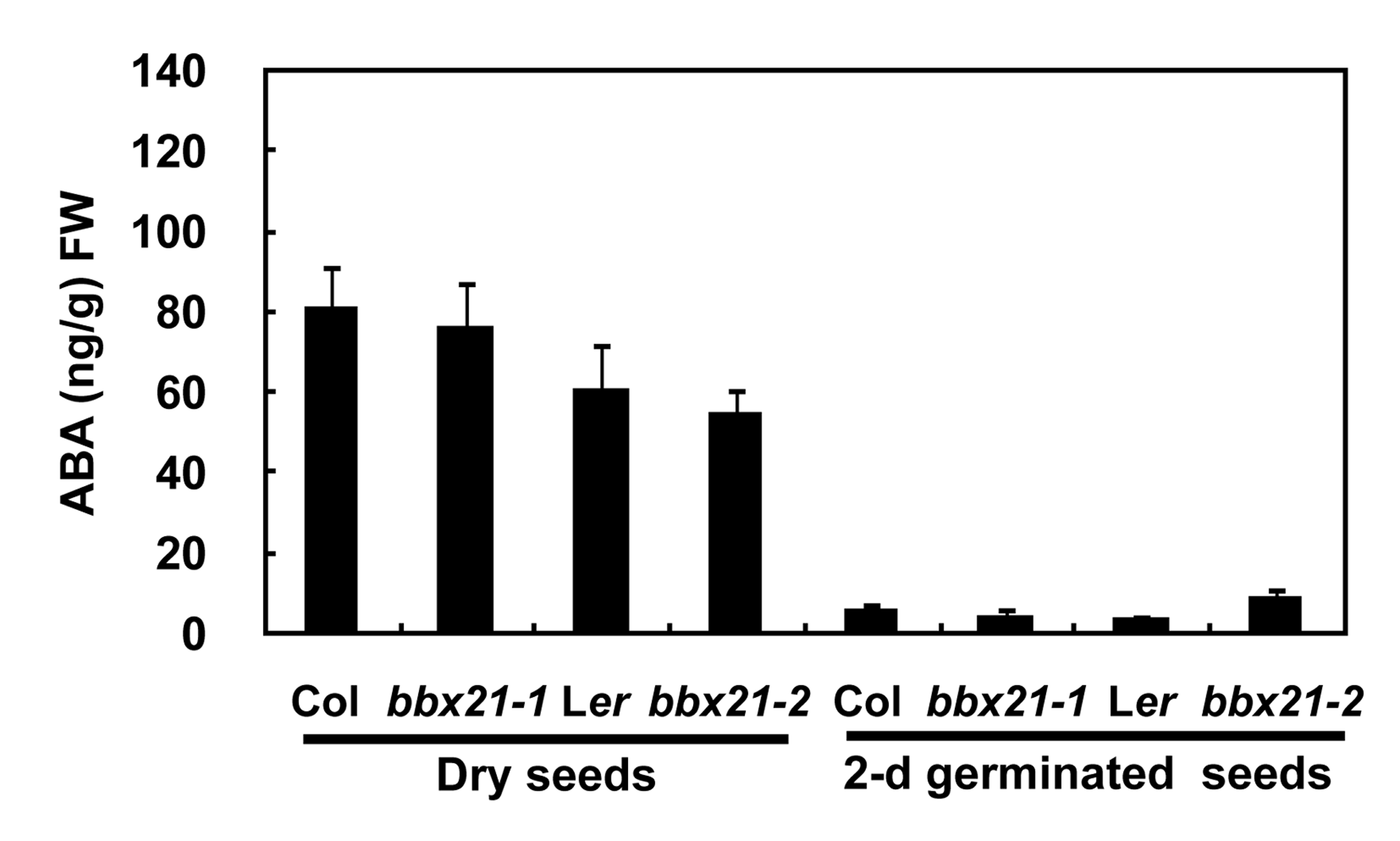

Supplement: Figure S1 — ABA levels in the dry or germinating seeds of the wild type and bbx21 mutants. Data are means of three independent experiments, and error bars represent SD. (TIF) [file pgen.1004197.s001.tif]

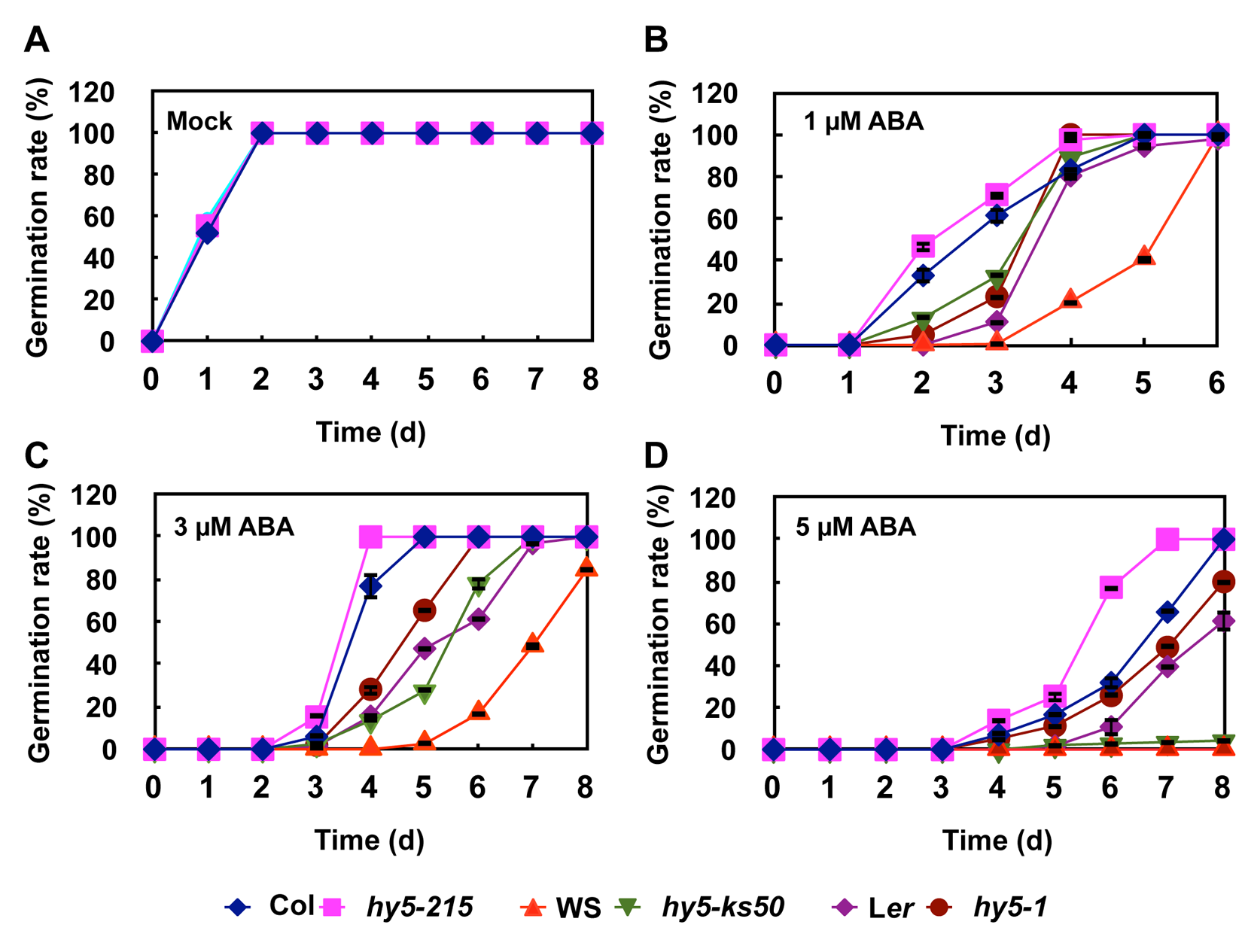

Supplement: Figure S2 — Germination rates of three hy5 mutants (hy5-215, hy5-ks50 and hy5-1) and their corresponding wild type controls under mock (A), 1 µM (B), 3 µM (C) and 5 µM (D) ABA treatments. (TIF) [file pgen.1004197.s002.tif]

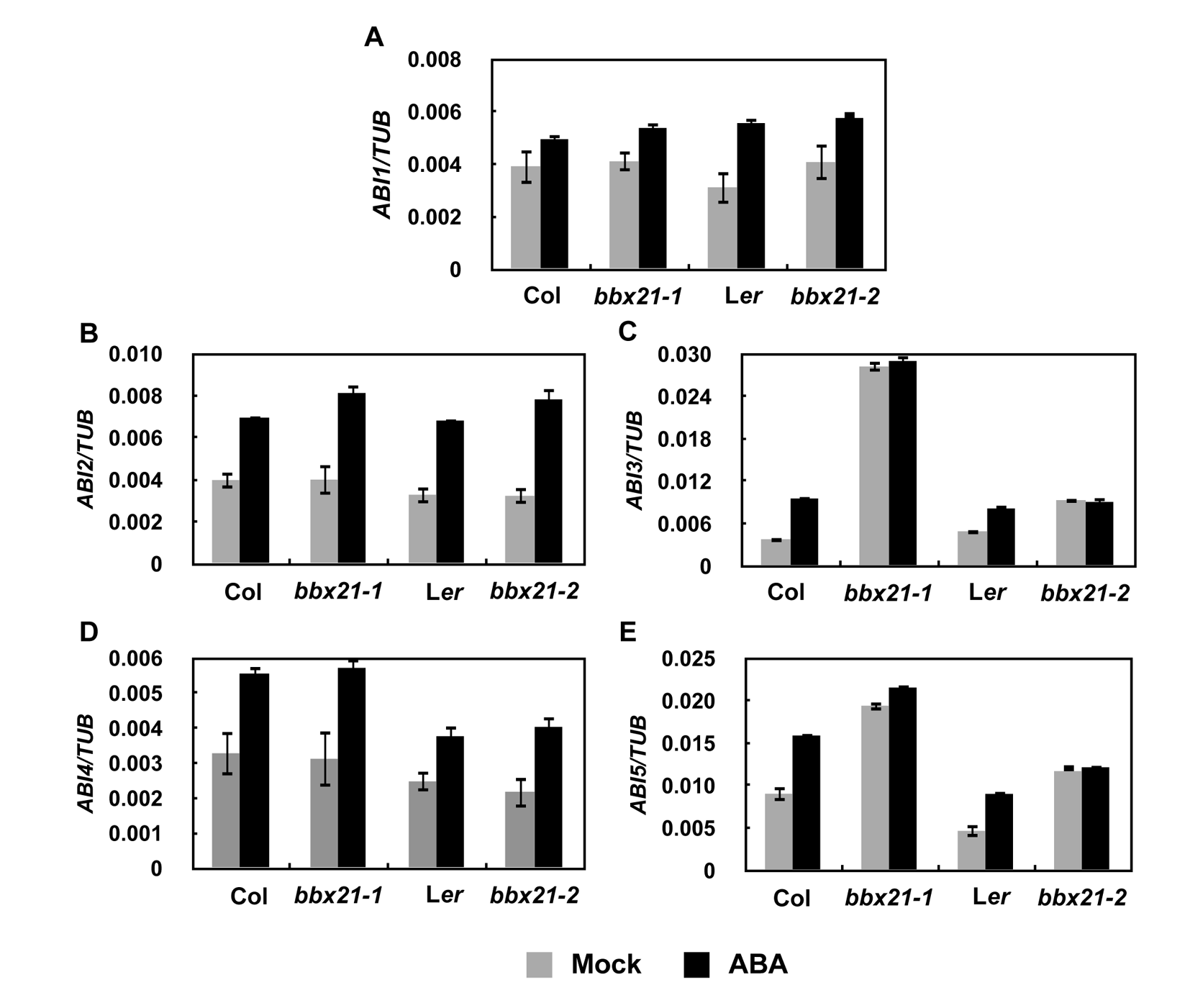

Supplement: Figure S3 — The expression levels of ABI1 (A), ABI2 (B), ABI3 (C), ABI4 (D) and ABI5 (E) in 2-d-old germinating seeds of bbx21-1 (Col) and bbx21-2 (Ler) mutants and their corresponding wild type controls treated with mock or 0.5 µM ABA. Data are means of three independent experiments, and error bars represent SD. (TIF) [file pgen.1004197.s003.tif]
