# Supplementary material for: Convergence of Light and ABA Signaling on the ABI5 Promoter
Source: PLoS Genet. 2014 Feb 27;10(2):e1004197. doi: 10.1371/journal.pgen.1004197 (PMC3937224; doi:10.1371/journal.pgen.1004197)
Supplement: Table S1 — A list of primers used in this study. (DOC) [file pgen.1004197.s004.doc]

Table S1: A list of primers used in this study.

| primer name | primer sequences (5’→3’)  (Note: The underlined nucleotides indicate the restriction sites for cloning.) | | |
| --- | --- | --- | --- |
| Genotyping | | | |
| abi2-1(F) | CATCATCTGCTATGGCAGG | | |
| abi2-1(R) | CCGGAGCATGAGCCACAG | | |
| abi3-1(F) | CGGTTTCTCTTGCAGAAAGTCTTGAAGCAAGTC | | |
| abi3-1(R) | TTGCCTCTAGCTCCGGCAAGT | | |
| abi4-101(F) | CCGAGAGCCACGTAAGCGCA | | |
| abi4-101(F) | CATCTGGACCATCTGATTTGGTG | | |
| abi5-1(F) | GGTTATTGTTGTGTATATGATGCAGTTG | | |
| abi5-1(R) | CCACTACTCTTTTCCTTCCCC | | |
| BBX21-LP | GGAACTACCGAACTATCATGGGCA | | |
| BBX21-RP | GAAGCCACCATCATCATACCA | | |
| DS5-1 | GAAACGGTCGGGAAACTAGCTCTAC | | |
| SALK_LBb1 | GCGTGGACCGCTTGCTGCAACT | | |
| Quantitative Real-time RT-PCR | | | |
| ABI1 (F) | TGAAGAAGCGTGTGAGATGG | | |
| ABI1 (R) | CTGTATCGCCAGCTTTGACA | | |
| ABI2 (F) | GATGGAAGATTCTGTCTCAACGATT | | |
| ABI2 (R) | GTTTCTCCTTCACTATCTCCTCCG | | |
| ABI3 (F) | CTGATTCTTGAATGGGTC | | |
| ABI3 (R) | TTGTTATTAGGGTTAGGGT | | |
| ABI4 (F) | CGTTAGGGCAGGAACAAGGA | | |
| ABI4 (R) | TAGAACATACCGGATCAACCAACG | | |
| ABI5(F) | GAGAATGCGCAGCTAAAACA | | |
| ABI5 (R) | GTGGACAACTCGGGTTCCTC | | |
| HY5 (F) | CCATCAAGCAGCGAGAGGTCATCAA | | |
| HY5 (R) | CGCCGATCCAGATTCTCTACCGGAA | | |
| BBX21(F) | GCACGGCCGACGAAGCATCT | | |
| BBX21(R) | TGTGTTCGTTCGCAGCGTGGA | | |
| Tubulin3 (F) | ATCCGTGAAGAGTACCCAGAT | | |
| Tubulin3 (R) | AAGAACCATGCACTCATCAGC | | |
| EMSA |  | | |
| ABI5-Probe | biotin-CCTTATGCAGTGAATAGTCCACGTGCACTCCCAATGGAAGTTCGGAATC  biotin-GATTCCGAACTTCCATTGGGAGTGCACGTGGACTATTCACTGCATAAGG | | |
| Yeast two-hybrid assay Plasmid Constructs | | | |
| ABI5-EcoRI (F) | | CCGGAATTCATGGTAACTAGAGAAACGAAGTTG | pAS-ABI5 |
| ABI5-NdeI (R) | | GGGAATTCCATATGGTAACTAGAGAAACGAAGTTGAC |
| BBX21-N-EcoRI(F) | | CCGGAATTCATGAAGATCAGGTGCGACGTCTG | pYX141-BBX21N |
| BBX21-N-HindIII(R) | | CCCAAGCTTAAGAAGAAACCTATCGTGTTTCTTTG |
| BBX21-C-EcoRI(F) | | CCGGAATTCACAGGGGTTAAGCTCTCTGCAAC | pYX141-BBX21C |
| BBX21-C-HindIII(R) | | CCCAAGCTTCCAGAAAGATCTAAACTTTTTATTAG |
| Yeast one-hybrid assay | | |  |
| ABI5- EcoRI(F) | | CCGGAATTCATGGTAACTA GAGAAACGA | pB42AD-ABI5 |
| ABI5-XhoI(R) | | CCGCTCGAGTTAGAGTGGACAACTCGGGTTCCTC |
| ABI5Pro-EcoRI(F) | | CCGGAATTCAGGCTTAGACAGCCATTAATTGTGAG | pLacZ2u-ABI5 pro |
| ABI5Pro-XhoI(R) | | CCGCTCGAGTTAACAACTGCATCATATACACAAC |
| ABI5pro-A-KpnI(F) | | CGGGGTACCATCTTGTGTTGATAAGTTCGC | pLacZ2u-ABI5 proA |
| ABI5pro-A-XhoI(R) | | CCGCTCGAGTTAACAACTGCATCATATACAC |
| ABI5pro-B-KpnI(F) | | CGGGGTACCTTGACCTTCACGCCTCTCTTCTT | pLacZ2u-ABI5 proB |
| ABI5pro-B-XhoI(R) | | CCGCTCGAGAAGCGAACTTATCAACACAAGAT |
| ABI5pro-C-KpnI(F) | | CGGGGTACCCTCCCAATGGAAGTTCGGAATC | pLacZ2u-ABI5 proC |
| ABI5pro-C-XhoI(R) | | CCGCTCGAGCTAAGAAGAGAGGCGTGAAGGTC |
| ABI5pro-D-KpnI(F) | | CGGGGTACCAGGCTTAGACAGCCATTAATTGTGAG | pLacZ2u-ABI5 proD |
| ABI5pro-D-XhoI(R) | | CCGCTCGAGCGTTGAGCTTCTAGCTGGTGAAAG |
| ABI5pro-G-box123-KpnI(F) | | CGGGGTACCCATTTGTGTAGCCGAAGTCAC | pLacZ2u-ABI5pro-G-box123 |
| ABI5pro-G-box123-XhoI(R) | | CCGCTCGAGGAATCCGTTCGGCTGCGGACGA |
| Protoplast Experiments | | | |
| ABI5Pro-EcoRI(F) | | CCGGAATTCAGGCTTAGACAGCCATTAATTGTGAG | pPCV814-ABI5pro |
| ABI5Pro-XhoI(R) | | CCGCTCGAGTTAACAACTGCATCATATACACAAC |
| ABI5-EcoRI(F) | | CCGGAATTCATGGTAACTAGAGAAACGAAGTTG | pRLT2-ABI5 |
| ABI5-BamH1(R) | | CGCGGATCCTTATTAGAGTGGACAACTCGGGTTCC |
| BBX21-EcoRI(F) | | CCGGAATTCATGAAGATCAGGTGCGACG | pRLT2-BBX21 |
| BBX21-BamH1(R) | | CGCGGATCCTTATCATTACCAGAAAGATCTAAACTTTTT |
| HY5-EcoRI(F) | | CCGGAATTCATGCAGGAACAAGCGACT | pRLT2-HY5 |
| HY5-BamH1(R) | | CGCGGATCCTTATCATCAAAGGCTTGCATCAGC |
| Protein expression | |  |  |
| BBX21-NheI(F) | | CTAGCTAGC ATGAAGATCAGGTGCGACG | pET28b-BBX21 |
| BBX21-NotI(R) | | AAGGAAAAAAAAGCGGCCGCTTATCATTACCAGAAAGATCTAAACTTTTT |
| HY5-BamHI (F) | | CGCGGATCC ATGCAGGAACAAGCGACT | pEGX-6P-1-HY5 |
| HY5-SalI (R) | | ACGC GTCGAC TTATCATCAAAGGCTTGCATCAGC |
